# Supplementary figures and images for: Pupil Dilation and Microsaccades Provide Complementary Insights into the Dynamics of Arousal and Instantaneous Attention during Effortful Listening
Source: J Neurosci. 2023 Jun 28;43(26):4856–66. doi: 10.1523/JNEUROSCI.0242-23.2023 (PMC10312051; doi:10.1523/JNEUROSCI.0242-23.2023)

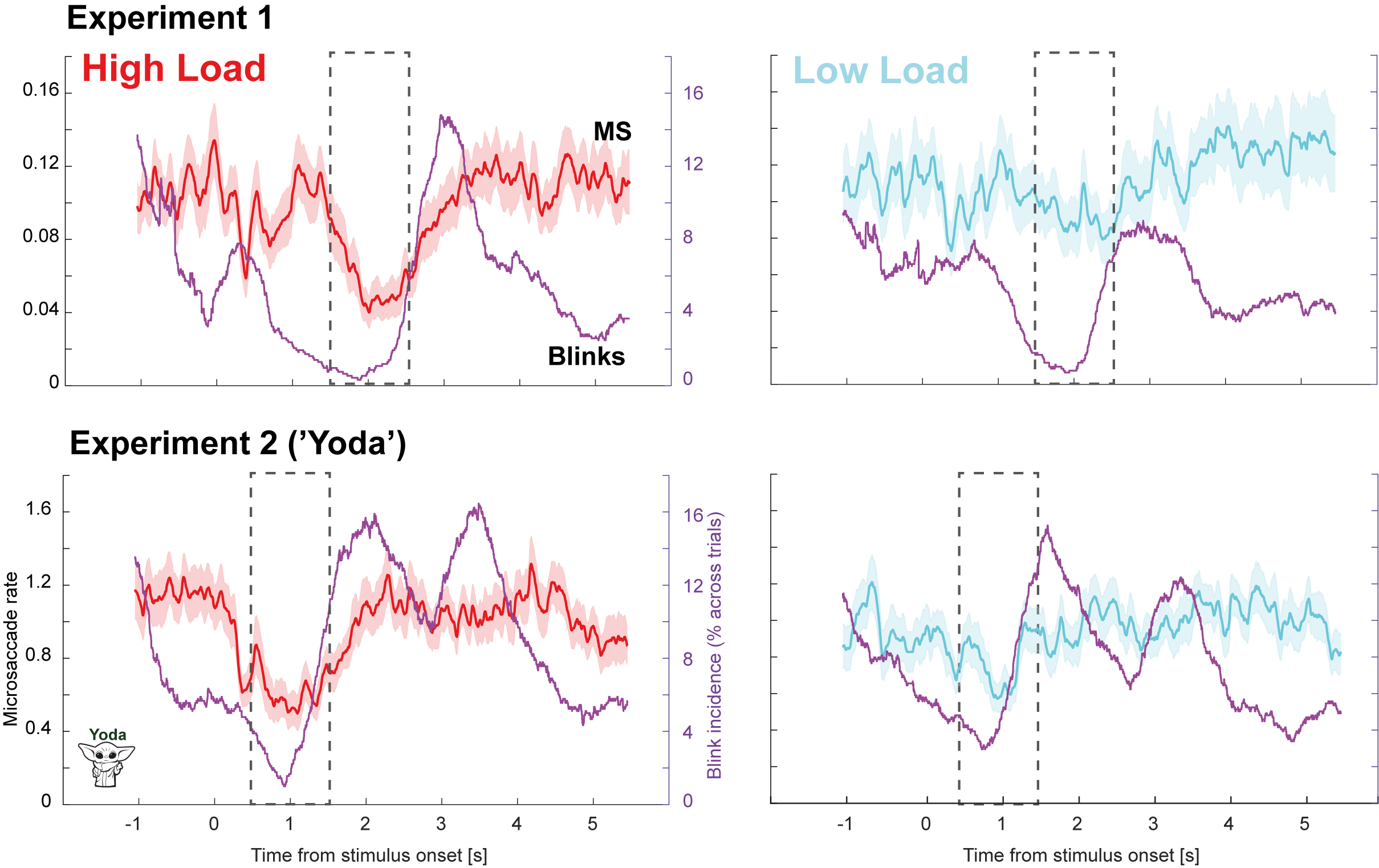

Supplement: Extended Data Figure 9-1 — MS rate modulation not explainable by increased blinking. MS rate from each experiment and condition (units on primary y-axis) is presented alongside associated blink rates (purple traces; units on secondary y-axis). Mean blink rates are computed by collapsing across trials and subjects and calculating incidence of “missing data” at each time point within the epoch. Results demonstrate reduced blinking rates during the critical intervals (where keywords are presented; dashed squares), confirming that MS rate reduction is not a consequence of blinking. Download Figure 9-1, TIF file. [file ns-JN-RM-0242-23-s01.tif]
